# A New Alloy of Aluminum

**Published:** 1869-02

**Authors:** 


A New Alloy of Aluminum, consisting of one-third silver and two-thirds of aluminum, has been introduced into the arts. It is said to be harder than silver, but more easily engraved.

